# PfRH5: A Novel Reticulocyte-Binding Family Homolog of *Plasmodium falciparum* that Binds to the Erythrocyte, and an Investigation of Its Receptor

**DOI:** 10.1371/journal.pone.0003300

**Published:** 2008-10-01

**Authors:** Marilis Rodriguez, Sara Lustigman, Estrella Montero, Yelena Oksov, Cheryl A. Lobo

**Affiliations:** 1 Laboratory of Blood-Borne Parasites, Lindsley Kimball Research Institute, The New York Blood Center, New York, New York, United States of America; 2 Laboratory of Molecular Parasitology, Lindsley Kimball Research Institute, The New York Blood Center, New York, New York, United States of America; 3 Electron Microscopy, Lindsley Kimball Research Institute, The New York Blood Center, New York, New York, United States of America; University of California Merced, United States of America

## Abstract

Multiple interactions between parasite ligands and their receptors on the human erythrocyte are a condition of successful *Plasmodium falciparum* invasion. The identification and characterization of these receptors presents a major challenge in the effort to understand the mechanism of invasion and to develop the means to prevent it. We describe here a novel member of the reticulocyte-binding family homolog (RH) of *P. falciparum*, PfRH5, and show that it binds to a previously unrecognized receptor on the RBC. PfRH5 is expressed as a 63 kDa protein and localized at the apical end of the invasive merozoite. We have expressed a fragment of PfRH5 which contains the RBC-binding domain and exhibits the same pattern of interactions with the RBC as the parent protein. Attachment is inhibited if the target cells are exposed to high concentrations of trypsin, but not to lower concentrations or to chymotrypsin or neuraminidase. We have determined the affinity, copy number and apparent molecular mass of the receptor protein. Thus, we have shown that PfRH5 is a novel erythrocyte-binding ligand and the identification and partial characterization of the new RBC receptor may indicate the existence of an unrecognized *P. falciparum* invasion pathway

## Introduction

P*lasmodium falciparum* infection is the most severe, and therefore most intensively studied form of human malaria [1]. The pathological consequences of infection derive from the parasitemia which develops through the cyclical asexual replication of parasites in the victim's red blood cells (RBC). Multiple parasite ligand-erythrocyte receptor interactions must precede a successful *Plasmodium* attack on the human red cell. The main steps in the invasion process are: (i) initial merozoite binding and reorientation, (ii) formation of a tight junction (the irreversible commitment of the parasite to invasion), and (iii) parasite entry [2].The parasite's ability first to recognize and then enter RBCs is central to infection and thus to the disease process, and consequently molecules involved in these steps are of especial interest for the development of malaria prophylaxis.

A combination of biochemical and biological studies, involving enzymatic modification of the RBC surface and the use of RBC variants, has clearly shown differences in the susceptibility of various RBCs to malarial invasion, and has pointed to some essential receptor-ligand interactions. The known receptors comprise all three major glycophorins A [3], B [4] and C [5], [6], as well as unidentified species, referred to as X [4], Y [7], Z [8] and E [9]. The reliance of the parasite on these RBC receptors differs between both laboratory [10]–[13] and field strains [14]–[16]. The ability of *P. falciparum* to exploit different receptors on the RBC for invasion represents an important mechanism that enables the parasite to respond to RBC polymorphisms and to evade the host immune system.

Two major malaria ligand families have been implicated in the various receptor–ligand interactions used by *Plasmodium* to invade human erythrocytes. The micronemal proteins form the *ebl* family (for ***e***rythrocyte ***b***inding ***l***igands) [17] and consist of 6 members, all containing a conserved cysteine-rich erythrocyte-binding domain at the N-terminal region, called the Duffy binding-like (DBL) domains, after region II of *P. vivax* Duffy-binding protein, the first functional RBC binding element to be identified [18].

A second family of erythrocyte binding proteins, the **R**eticulocyte binding **H**omolog (Pf*RH* family) in *Plasmodium* was initially found in the rodent malaria *P. yoelii* (the Py235 family) [19] and was implicated in the ability of this parasite to invade mature mouse RBCs. Related reticulocyte binding proteins, PvRBP-1 and 2 [20] were subsequently identified in *P. vivax*. Functional genomics analysis of the *P. falciparum* database yielded the PfRH family, consisting of six genes (*PfRH1*, *PfRH2a*, *PfRH2b*, *PfRH3*, *PfRH4 and PfRH5*), which share homology and structural features with both the *P. yoelii* and *P. vivax* families [8], [21], [22]. The PfRH1 protein of the RH family has been shown to bind erythrocytes in a sialic-acid-dependent manner [7]. In contrast, PfRH2b has not been demonstrated to bind directly to red cells; however, targeted gene disruption has shown that it is required for a sialic-acid-independent invasion pathway [8]. Recently, Gaur et al reported the putative role of PfRH4 in parasite invasion via binding to a neuraminidase-resistant receptor [23]. All PfRH proteins except *PfRh3* [24] are expressed in merozoites and are located at their apical end, consistent with a role in ligand–receptor interactions [8].

The *PfRH5* gene in *P. falciparum* was identified from the *P. falciparum* genome sequence [21] (PlasmoDB), but the putative protein encoded by this gene has not been analyzed, nor has its role in parasite invasion been explored. Additionally, an earlier report alluded to the essential role of PfRH5 in invasion, as attempts to disrupt *PfRH5* failed [21]. This lent support to the need for a detailed functional study of this molecule. In this report, we characterize this final member of the PfRH family. We demonstrate that PfRH5 binds to the surface of erythrocytes; that its target is a sialic-acid-independent receptor; that this receptor is sensitive to high concentrations of trypsin; and that a 143-aa recombinant fragment of PfRH5 binds to the RBC with the same specificity as the intact protein. We have further characterized its interaction with the RBC in terms of the stoichiometry (number of copies per red cell) and affinity and obtained a molecular mass for the putative receptor. Our results thus imply the involvement of a novel RBC receptor molecule in *P. falciparum* invasion, and reveal its abundance on the membrane and its affinity for the parasite ligand.

## Results

### PfRH5 is a novel member of the RH family

**Figure 1:**
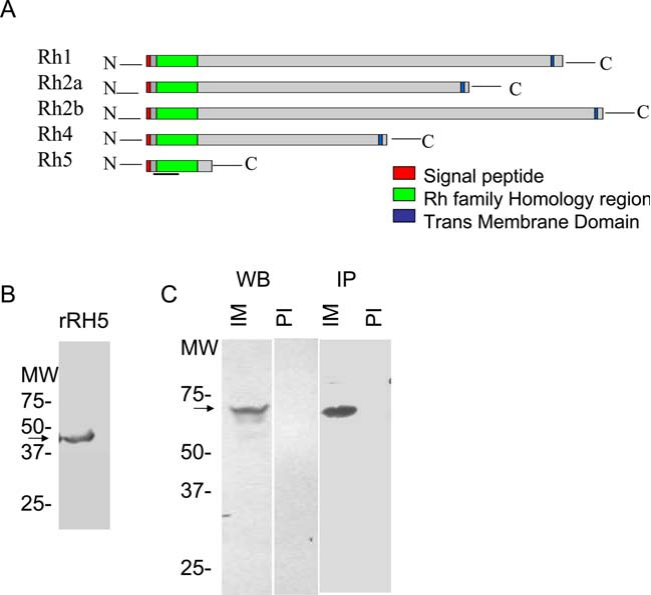
Cartoon of PfRH ligands and characterization in the parasite. A. Schematic depiction of different members of the PfRH family, showing location of the signal peptide, region of homology among the various RH ligands and the trans-membrane region at the C-termini of the proteins. The bar at the bottom of PfRH5 marks the region of PfRH5 that was expressed in *E. coli*. B. Expression of a recombinant 43-kDa protein of PfRH5 (rRH5), chosen on the basis of homology with putative binding domains of *P. vivax* reticulocyte-binding protein 1 (PvRBP1) and PfRH4. Arrow indicates rRH5 after purification on GST-agarose column. C. Western Blot and immunoprecipitation analysis of asynchronous Dd2 parasite lysates with anti-rRH5 antibodies (IM). Pre-immune serum (PI) was used as a negative control. Arrow indicates specific 63 kDA RH5 protein seen in lysates.

Ligands belonging to the reticulocyte-binding protein family, found in different *Plasmodium* species, are high MW proteins that share a low level of amino acid homology and structural features, notably a short exon 1 encoding a signal sequence, followed by a large exon 2 encoding the bulk of the protein, and a single predicted transmembrane domain (TMD) close to the cytoplasmic COOH terminus (Fig. 1 A) [25]. The gene (PFD1145c) encoding the ligand PfRH5, described in this paper, is not a typical member of this family, being relatively small and lacking the transmembrane and cytoplasmic domains (Fig. 1A). The cDNA is composed of only 1581 bp and encodes a putative polypeptide of 526 amino acids with a calculated molecular mass of 63 kDa. CLUSTAL W alignments of the predicted PfRH5 amino acid sequence with the other RH members support a familial relationship, with an overall level of similarity of 15–30% (identity plus conservative substitutions) to the different RH members [http://plasmodb.org].

### PfRH5 is expressed as a 63 kDa protein in the asexual parasite

The members of the PfRH protein family have no obvious domain structures, such as the Region II cysteine-rich domains in the EBL family. However, the N-terminal amino acids of the PfRH ligands are the most highly conserved in the sequences of these proteins. Additionally, this region contains most of the proteins' cysteine residues, which could reflect the presence of binding sites. Thus, we focused on the N-terminus of PfRH5 and chose a 143-aa sequence of PfRH5 from Asn-31 to Val-174 on the basis of a Clustal alignment of PfRH5 with the phylogenetically close PvRBP1 [20] (∼22% overall similarity, data not shown). This region of RH5, moreover, also exhibited significant homology with the RBC-binding domain of PfRH4 [23]. The recombinant protein was expressed as a GST fusion protein of ∼43 kDa in *E. coli* and purified to homogeneity, using glutathione-Sepharose 4B (Fig. 1 B). Antiserum raised in rabbits against the purified protein was used to identify native PfRH5 in asynchronous parasite extracts, by immunoprecipitation and immunoblotting. Fig. 1 C shows the results of these assays, in which a 63 kDa protein is clearly seen in the 3D7 parasite extracts, the position corresponding to the predicted molecular mass of PfRH5. The protein does not appear to undergo processing, as no lower MW bands are apparent on either the blot or the autoradiograph. Similar results were obtained from parasite extracts from Dd2 and HB3, indicating that all these strains synthesize PfRH5, with no perceptible difference in size (data not shown).

### PfRH5 is expressed in the rhoptries of merozoites

**Figure 2:**
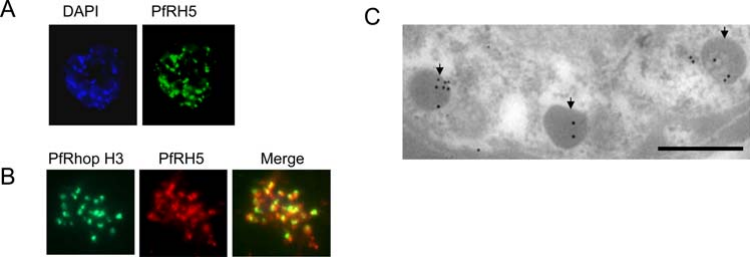
Apical localization of PfRH5 in the merozoite. A Mature Dd2 schizonts were labeled with rabbit anti-PfRH5 IgG and counterstained with FITC-labeled anti-rabbit IgG (green) using immunofluorescence microscopy. B Colocalization studies: Mature Dd2 schizonts were double-labeled with anti-PfRH5 IgG (red) and anti-RhopH3 (green) monoclonal antibody. The merged staining (yellow) by both antibodies indicates their similar location within the parasite. C Immunoelectron microscopy confirming the localization of PfRH5 in the rhoptries within the developing merozoites in the Dd2 schizont. Parasites were fixed with 1% paraformaldehyde and 0.1% glutaraldehyde. Staining was detected with rabbit anti-RH5 and anti-rabbit gold (10 nm). Scale bar represents 500 nm.

All the PfRH proteins that have been described so far have been localized to the apical, invasive end of the merozoite. This should be true of PfRH5 if it plays a role in parasite entry. The subcellular localization of RH5 was determined by immunofluorescence using anti-RH5 rabbit antibodies. Fig. 2A shows the staining pattern obtained on late-stage schizonts demonstrating that RH5 is indeed located at the apical end of merozoites. The punctate staining in this region, wherein a single dot occasionally resolves into double foci, is a hallmark of rhoptry localization. Co-staining of late stage schizonts was then performed, using antibodies against a known rhoptry marker, PfRhopH3 [26]. Fig. 2 B shows the results of this dual IFA, and reveals a partial overlap of staining patterns by the two antisera. Thus, although both RhopH3 and PfRH5 may be present in the rhoptries, they may be located in different parts of the rhoptry.

We confirmed this specific localization of PfRH5 in rhoptries by immuno-electronmicroscopic analysis (IEM). The IEM was carried out on sections of schizont-infected RBC with anti-PfRH5 antibodies. As can be seen in Fig. 2C, discrete antibody reactivity was observed in the electron-dense organelles with the morphological characteristics of rhoptries (indicated by arrows in Fig. 2C). Thus, like the other RH ligands, PfRH5 is localized within the rhoptries, strongly suggesting a role in merozoite invasion.

### PfRH5 binds to erythrocytes

**Figure 3:**
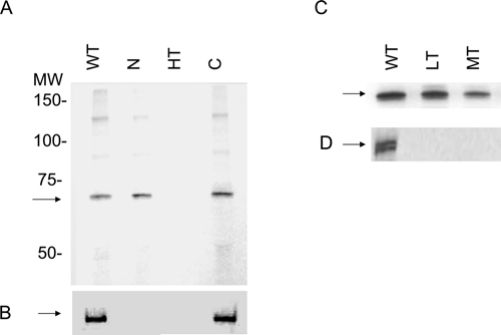
Erythrocyte-binding activity of native PfRH5. A Binding of the native PfRH5 protein in the HB3 culture supernatant incubated with untreated (WT) erythrocytes, variously enzyme-treated erythrocytes (N: neuraminidase; HT, high trypsin; C, chymotrypsin). The PfRH5 parasite protein was detected in the eluate fractions by immunoprecipitation with anti-RH5 antibodies in WT, N and C lanes but not HT. B The same eluate samples from (A) were used for the detection of EBA-175 by immunoprecipitation. EBA-175 binds to wild type and chymotrypsin-treated erythrocytes but not to neuraminidase and high trypsin-treated erythrocytes C Binding of the native RH5 protein to erythrocytes treated with lower concentrations of trypsin (LT low trypsin and MT: moderate trypsin). D The eluate samples from (C) were used for the detection of EBA-175 by immunoprecipitation. EBA-175 binds to untreated erythrocytes but not to low-trypsin- and moderate-trypsin-treated erythrocytes.

Having established its location at the invasive apical end of the merozoite and homology with other known RBC binding proteins, we performed assays to determine whether native PfRH5 binds to RBCs. The native full-length PfRH5 was isolated from [^35^S]methionine/cysteine-labeled culture supernatants (HB3 strain) that contained merozoites released from infected erythrocytes in the absence of target erythrocytes. Studies have shown that extracellular merozoites release parasite proteins into the culture, and such culture supernatants can be a source of parasite ligands that bind erythrocytes. Thus, [^35^S]methionine/cysteine-labeled spent merozoite supernatants were used as the source of RBC-binding proteins, and when the eluate was immunoprecipitated with anti-RH5 antiserum, a dominant band at ∼63 kDa was seen (Fig. 3A, lane WT). Thus, PfRH5 appears to be an adhesin that participates in invasion by binding to the RBC surface.

### The RBC receptor for PfRH5 is resistant to neuraminidase, low and moderate trypsin and chymotrypsin, but sensitive to treatment with high trypsin

Treatment of RBCs with enzymes that selectively cleave moieties of surface proteins, followed by an analysis of the resulting effects on ligand binding, afforded a first indication of molecules that could serve as receptor(s) for PfRH5 during invasion. RBCs were treated with neuraminidase, high trypsin concentration, and chymotrypsin, and used for EBAs with radio-labeled HB3 parasite supernatant in several independent experiments to detect the receptor recognized by native PfRH5. Fig. 3A shows the RBC receptor profile for PfRH5. PfRH5 binding was unaffected by neuraminidase (N) and chymotrypsin (C) (Fig. 3 a, lanes N and C), but was eliminated by treatment with high concentrations of trypsin (lane HT: 10 mg/ml), indicating that adhesion is dependent on trypsin-sensitive sites on the RBC surface. Most malaria ligand binding assays employ lower levels of trypsin (up to 1.5 mg/ml) to denude the RBCs of tryspin-sensitive moieties. However routine procedures in immunohematology labs, including that of the New York Blood Center (Reid M, personal communication), call for a more stringent treatment of the red cell with 10 mg/ml of trypsin for the same time period of treatment, to ensure complete elimination of all trypsin-sensitive molecules, besides GPA and GPC. Low and moderate concentrations of trypsin (LT, 0.5 mg/ml; MT, 1.5 mg/ml) did not impede PfRH5 binding (Fig. 3 C). In contrast, EBA-175 binding was ablated by treatment of the RBC with trypsin at all concentrations, confirming that the low trypsin levels sufficed to cleave glycophorin A residues (Fig. 3 B and 3D). Thus, PfRH5 binds to RBCs in a sialic-acid-independent fashion, similar to PfRH4 but unlike PfRH1. However, the PfRH4 receptor exhibits sensitivity to low trypsin and chymotrypsin, unlike that of PfRH5, and thereby distinguishes itself from the RBC molecule recognized in our assay. Thus, the binding profile of native RH5 to the RBC suggests the participation of a novel red cell receptor in merozoite invasion.

### rRH5 Binds erythrocytes, yielding a similar receptor profile

**Figure 4:**
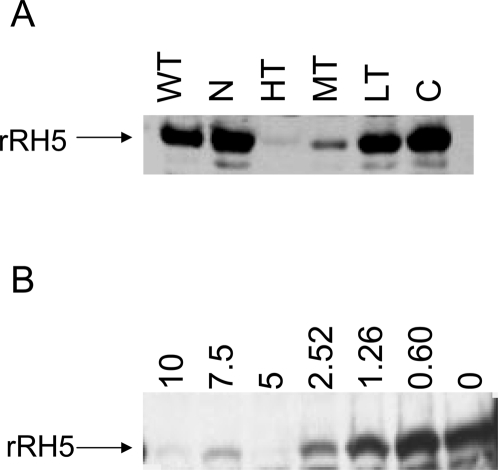
Erythrocyte-binding activity of rRH5. A 0.6 µmoles of rRH5 were used in binding assays with untreated RBCs and RBCs treated with neuraminidase- (N), low-trypsin- (LT), medium-trypsin- (MT), high-trypsin- (HT) and chymotrypsin, followed by elution and immunoprecipitation with anti-RH5 antibodies. Gels containing the immuno-precipitates were blotted and probed with anti-RH5 antibodies. B Antibodies to rRH5 block the erythrocyte binding of the recombinant protein. Total IgG from rabbits immunized with rRH5, blocks erythrocyte binding of the RH5 recombinant protein. 0.6 µmoles of rRH5 was incubated with normal erythrocytes in the presence of purified IgG from rabbit sera at final concentrations of 0–10 µg/100 µl.

To delineate the binding region on PfRH5, a 143-aa fragment of RH5 was assayed for erythrocyte binding. This sequence was chosen because a similar homologous region in another RH homolog, PfRH4 was found to bind RBCs [23]. The specificity of the interaction of rRH5 with the erythrocytes was studied in several ways:

- The binding of rRH5 to wild type and various surface-modified erythrocytes was compared with the binding of the native PfRH5: as can be seen in Fig. 4 A the binding profile of rRH5 simulates that of native RH5, in that it binds to a receptor that is non-sialylated but sensitive to high concentrations of trypsin. Low concentrations of trypsin (0.5 mg/ml) had no inhibitory effect on the binding, while moderate (1.5 mg/ml) trypsin concentrations visibly decreased the binding. Thus rRH5 appears to bind with the same specificity as the native PfRH5, indicating that rRH5 contains an intact receptor-binding domain, as in the native protein. This result permits the use of rRH5 in place of the native parasite supernatant for further analysis of its interaction with the RBC.
- To establish the specificity of the binding, the capacity of anti-RH5 antibodies to inhibit attachment of the rPfRH5 to erythrocytes was tested. rRH5 was incubated with wild type erythrocytes in the erythrocyte binding assay, in the presence of different amounts of purified anti-RH5 IgG, from 0–10 µg IgG. Fig. 4B demonstrates specific inhibition of rRH5 binding, as with increasing amounts of antibody progressively lower amounts of rRH5 were detected in the binding eluate. This confirmed the specificity of binding of rRH5 to the RBC.

### Characterization of the interaction of rRH5 and the RBC

We undertook a quantitative analysis of the interaction between RH5 and the RBC. The affinity of the interaction of RH5 with its receptor, and the receptor titer on the RBC may shed light on the identity of the receptor, and could also define the role of RH5 in the hierarchy of molecular interactions needed for successful invasion.

**Figure 5:**
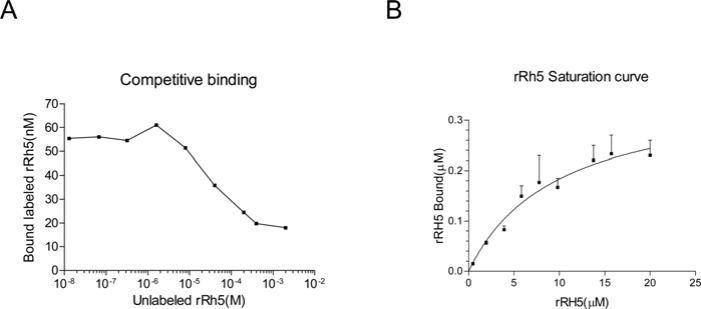
Characterization of binding kinetics of rRH5 and RBC. A Competition of binding to RBCs of rhodamine-labeled against unlabeled rRH5. Binding is inhibited by unlabeled rRH5 in a dose-dependent manner. The inhibition curve derived from scans of two independent assays shows that rRH5 competes against the binding of labeled rRH5 at an IC_50_ of ca. 5×10^−5^ M. B Binding profile of rRH5 to resealed RBC ghosts. The curve is the calculated least-squares best fit for a population of identical independent binding sites, giving K_d_ = 1.25 (±3)×10^−5^ M, with a saturation level (number of sites) at 0.42 µM.

A competitive binding assay was set up by mixing labeled and unlabeled rRH5 using increasing amounts of the unlabeled ligand, to give total concentrations of 0.1 to 400 µM with 25 µl of packed ghosts in a total volume of 100 µl. As Fig. 5A shows, the unlabeled protein competes against the labeled protein, with a concentration for 50% inhibition of ca. 5×10^−5^ M, implying a slightly smaller affinity (ca threefold increase in K_d_). Thus specificity of binding is retained after labeling, and the affinity is almost unperturbed. Similar experiments with rhodamine-GST did not reveal any binding to the ghosts (data not shown).

We accordingly performed binding assays of the rhodamine-labeled rRH5 to resealed RBC ghosts. Varying amounts of rRH5 were incubated with a fixed concentration of ghosts, and bound polypeptide was determined by fluorimetry on the ghosts, dissolved in SDS. The typical parabolic binding profile shown in Fig. 5B, delivers a dissociation constant of 12 (±3) µM.

From the number of ghosts in the assay mixture and the saturation binding concentration corresponding to the best fit to the profile (0.42 (±0.06) µM) we can calculate a copy-number for the receptors on each cell which works out to ∼10^5^. This value enables us to exclude the most abundant RBC surface-exposed proteins, band 3 (copy number 1.2×10^6^ per cell), and glycophorin A (8×10^5^ copies), as well as candidates of low abundance, such as the Kell protein (4000–18,000).

### Overlay assays identify a ∼32 kDa RBC protein that binds to RH5

**Figure 6:**
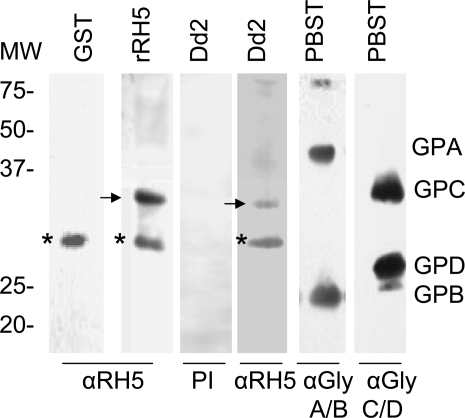
PfRH5 binds to a ∼32 kDa protein on human erythrocytes. Normal erythrocyte ghost cells were separated by SDS–PAGE, and the gel was blotted and incubated with GST (the fusion partner of rRH5) or rRH5 or native parasite culture supernatant. After extensive washing, bound protein was detected by rabbit anti-RH5-GST and blots were processed by enhanced chemiluminescence (ECL, Amersham Biosciences). A specific target protein of ∼32 kDA is seen in rRH5 lane (marked with arrow) and Dd2 lanes (marked with arrow). Asterisk denotes a non-specific protein band that appears in control GST and other lanes. Parallel blots were probed with anti-GPA/B or anti GPC/D antibodies to define positions of the glycophorins relative to the 32 kDA band. Pre-immune rabbit sera did not yield any reactivity (PI).

To identify the receptor(s) of PfRH5 on human erythrocytes, we used gel overlay experiments in which erythrocyte ghost proteins, separated on SDS-PAGE, were incubated with rRH5 (Fig. 6, rRH5) or with Dd2 culture supernatant (Fig. 6, Dd2). As a control, GST, the fusion partner pf rRH5.was included in the overlay assay (GST). After exposure of the ghost proteins on the membrane to these three different antigens the blot was treated with anti-RH5 (which contains antibodies to GST too) to detect specific ligand-receptor interactions. A non-specific ∼28 kDa RBC protein (Fig. 6, marked by asterisk) was seen in all the lanes. However, we were able to detect a specific interaction between rRH5 and a component of apparent molecular mass of 32 kDa on the blot of ghost proteins (indicated by arrow, lane rRH5). The same 32 kDa band was seen in the lanes treated with native parasite supernatant (Dd2, indicated by arrow). Thus, the 32 kDa erythrocyte protein interacts with rRH5 as well as the native RH5 from the parasite supernatant. Pre-immune rabbit sera did not react with any of the proteins on the ghost overlay (PI). To determine whether any of the glycophorins migrated at this position on the blot, parallel strips were treated with anti-GPA/B and anti-GPC/D. The 32 kDa band does not migrate near any of the glycophorins (lanes GPA/B and GPC/D), thus, it appears, ruling them out as PfRH5 receptors. Future work will be directed at identifying this putative ∼32 kDa receptor molecule.

### Anti-RH5 does Not inhibit merozoite invasion

Since RH5 appeared to play a role in invasion by binding to the RBC, we conjectured that antisera against rRH5 might inhibit parasite entry . Unexpectedly, however, no significant inhibitory effect (∼4.5% after subtracting the inhibition obtained with pre-immune) was seen on merozoite invasion of untreated RBCs when purified anti-RH5 IgG was added to the invasion assay (Table 1, WT, WT+Ab). We next decided to assess the effects of the antibody on invasion into enzyme-treated red cells (Table 1). Red cells were treated with neuraminidase, trypsin and chymotrypsin and invasion assays were performed in the presence or absence of 0.5 mg/ml purified anti-rRH5. Inhibitory effects were assayed by comparing the extent of inhibition caused by the antibody relative to basal invasion efficiency observed in host cells treated with the same enzymes. Additionally, the effect of the antibodies was compared to those engendered by the pre-immune sera. Anti-RH5 antibodies had no effect on the invasion of the parasites into neuraminidase-treated RBCs (Table 1, N+Ab vs N+PI). In trypsin-treated RBCs we found a small decrease in invasion efficiencies in the presence of the anti-RH5 antibody, compared to the pre-immune (6.6%) that was not found to be statistically significant. No inhibitory effect was seen in chymotrypsin-treated RBCs. Thus, anti-RH5 does not exert a significant inhibitory effect on parasite invasion. These same antibodies, however, did inhibit the binding of rRH5 to the RBC. Thus, it appears that although RH5 binds RBCs, the ligand-receptor complex is not accessible to the antibody during invasion. Alternatively, since the antibody was directed against a small portion of the full-length RH5, it may not occlude invasion-significant epitopes. All these invasion assays were carried out with the Dd2 strain of the parasite, and future work will be directed at assessing inhibition in other parasite strains.

**Table 1:** Growth inhibition assay (GIA) of the anti-PfRH5 IgG against the Dd2clone of *P. falciparum*.

RBC	% Parasitemia	% Inhibition
Untreated RBC	4.1±0.20	—
Untreated+Ab	3.6±0.20	12
Untreated+PI	3.8±0.60	7.5
Neuraminidase Treated	1.4±0.04	—
Neuraminidase Treated+Ab	1.4±0.04	0
Neuraminidase Treated+PI	1.6±0.20	−15
Trypsin Treated	3.2±0.04	—
Trypsin Treated+Ab	2.7±0.10	16
Trypsin Treated+PI	2.9±0.40	9.4
Chymotrypsin Treated	2.2±0.19	—
Chymotrypsin Treated+Ab	2.1±0.05	4.5
Chymotrypsin Treated+PI	2.3±0.10	−4.5

Ab: Anti-RH5, PI: pre-immune sera.

## Discussion

Erythrocyte invasion by *P. falciparum* merozoites is a highly complex and incompletely understood process. A large multi-molecular cascade of interactions between red cell receptors and parasite ligands is hypothesized to be involved in the sequence of steps. However, only some of these molecules are key players, such as can allow the parasite to maintain adequate invasion potential despite physiological changes in host RBCs or immunological alterations in the host. This in turn will allow the persistence of parasites in the host and ultimately increase the success of malaria transmission. These key molecules are most likely specific parasite adhesin molecules, involved in the recognition and binding events at the RBC surface that lead to eventual entry. In this paper we present one such parasite ligand, PfRH5, the smallest member of the PfRH family, which despite lacking a transmembrane and cytosolic region at the C-terminus, shows significant structural and sequence homology to the RH family (Figure 1A). The overall gene sequence homology between the different RH family members is low, but all contain a number of conserved blocks of amino acids which clearly identify them as members of this ligand family. The RH family of ligands has been postulated to play a role in determining what RBC receptors are used during invasion [8] and thus are expected to bind to the red cell.

Surprisingly, of the four RH ligands characterized so far, only two have been shown to directly bind to the red cell surface, PfRH1 to a neuraminidase- and chymotrypsin-sensitive receptor [7], and PfRH4 to a neuraminidase-resistant but trypsin- and chymotrypsin-sensitive molecule on the RBC [23] . We have established that the receptor(s) recognized by PfRH5 contain no sialic acid residues and are resistant to chymotrypsin and to low levels of trypsin (<1.5 mg/ml), but sensitive to high levels of trypsin (10 mg/ml). Thus, our results point to the involvement of a new type of RBC receptor. We can rule out the involvement of the major RBC sialoglycoproteins, the glycophorins A, B and C, since (1) they are all highly sialylated molecules and therefore neuraminidase-sensitive, and (2) our overlay assay points to a −32 kDa RBC protein as a candidate for the interaction, with which none of the glycophorins conforms. Two molecules that fit the size criterion and the sialic acid-independent nature of the receptor are the Rh blood group proteins and the unglycosylated form of the RBC aquaporin. The copy number of the Rh blood group proteins is known to vary substantially among individuals ranging from 10,000 to 200,000 molecules/RBC, while the aquaporin is found at around 150,000 molecules per RBC [27]. Further work with cells lacking these various candidate receptor molecules in binding and invasion assays is needed for a conclusive identification of the receptor.

Studies on the PfRH ligands have shown that different parasite lines exhibit significant sequence variation between the same family members, ranging from a few amino acid changes to large deletions [24], [28]. It has also been reported that some PfRH ligands are missing in some strains [8] . These sequence variations might represent a type of antigenic diversity and/or govern changes in the binding properties of the protein. The latter explanation is supported by our recent study on Brazilian field isolates, which associates different polymorphism in the RH ligands with changes in the invasion pathways used by the parasite [28]. Further complexity arises from the fact that the transcription and expression patterns of PfRH ligands vary between different parasite lines [8], [24]. These variations have implications in the way the proteins are utilized, for changes in the expression of PfRH ligands could lead to the recognition of different receptors on the erythrocyte surface and therefore merozoites with different invasion potentials. Similar sequence analyses of PfRH5 among different laboratory and field strains of the parasite may shed light on the utilization of this new RH family member in invasion, and on varying patterns of RBC receptor usage.

Cowman and Crabb had earlier reported an inability to disrupt the PfRH5 gene [21]. Thus, it appears that PfRH5 must have a critical function in parasite survival. Our present data, based on antibodies against RH5, show a lack of significant inhibitory effect on parasite invasion. This was true even when the parasite underwent invasion selection, as evidenced by invasion assays in RBCs treated with different enzymes. Thus, it appears that the redundancy in erythrocyte invasion ligands of *Plasmodium*, allied to the large repertoire of parasite receptors belonging to the DBL and RH families, had been exploited leading to this absence of inhibition of invasion by anti-PfRH5. This would not, on the other hand, explain why, by contrast with the other PfRH family members, *PfRH5* could not be disrupted.

In summary, we have identified a novel malaria red cell binding ligand, which is expressed at the invasive apical end of the merozoite, and which, on the basis of gene structure and amino acid homology, belongs to the PfRH family. We propose that PfRH5, as previously shown for its orthologs, plays an important adhesion function by binding to a previously unrecognized type of receptor on the RBC. Further study of this molecule across different laboratory and field strains of *P. falciparum* may provide insights into the essential features of this critical component of the invasion cascade, and stimulate the search for a possible role in developing new malaria vaccines.

## Materials and Methods

### P. falciparum parasites

*P. falciparum* strains 3D7, HB3, and Dd2 were obtained from the Malaria Research and Reference Reagent Resource Center (MR4) and were cultured in human A^+^ erythrocytes [29]. The identity of each strain has been confirmed by microsatellite fingerprinting [30].

### Expression of the recombinant PfRH5 Protein (rRH5) and production of anti-RH5

The amino acid sequence of the PfRH5 protein in the 3D7 parasite clone was used to express a fragment of PfRH5, constituting 143 aa from Asn-31 to Val-174. A 429-bp fragment of the *PfRh5* gene encoding the 143 aa was amplified by using the following primers: primers 5′-CGC GGA TCC AAA ACG AAG AAT CAA-3′ and 5′-CCG CTC GAG AAA ATC CAA ATG TCC TTC-3′. The PCR product was digested with BamHI and Xho1 (New England Biolabs, Beverly, MA) and inserted downstream of the T7 promoter in the *E. coli* expression vector, pGEX (Amersham, Pharmacia Biotech), to obtain the plasmid pRH5-pGEX. *E. coli* BL21(DE3) cells (Novagen, San Diego, CA) were transformed with pRH5-pGEX and used for the expression of rRH5. Soluble fusion protein was purified from *Escherichia coli* using standard methodologies using glutathione-Sepharose 4B and injected into New Zealand White rabbits using 50 µg of rRH5 on days 0, 28, 56, and 84. The rabbit sera were collected on day 98 and were used in all the following experiments.

### Biosynthetic radiolabeling and immunoprecipitation

Biosynthetic radiolabeling of asynchronous intraerythrocytic parasites with [^35^S] methionine/cysteine (ICN) was performed at 100 µCi/ml during *in-vitro* culture. Labeled parasites were lysed on ice for 30 min in PBS with 1% Triton ×100 and standard protease inhibitors, added with occasional vortexing. Lysates were clarified by centrifugation. Immunoprecipitations with pre-immune or anti-RH5 were performed as described [5].

### Immunoblotting

Saponin-lysed pellets from asynchronous parasites were separated on 10% SDS-PAGE gels under reducing conditions and blotted onto nitrocellulose membranes. Rabbit anti-RH5 and appropriate secondary antibody were used to detect specific immuno-reactivity. Pre-bleeds from the same rabbits were used as pre-immune controls.

### Immunofluorescence and immuno-electronmicroscopic localization of PfRH5

Schizont-enriched parasites were smeared on slides and stored at −80°C. Slides were thawed, acetone-fixed, reacted with anti-RH5 at room temperature for 1 h, and then reacted with FITC-conjugated goat anti-rabbit antibody (1∶200) at room temperature for 30 min. The FITC-stained images were examined with a fluorescence microscope and recorded using Adobe Photoshop. For co-localization studies, late-stage schizonts were double-stained with rabbit anti-PfRH5 and 1B9 (MAb anti-RhopH3) [26], followed by rhodamine-conjugated anti-rabbit antibody (1∶200) and FITC-conjugated anti-mouse antibody (1∶200). All smears were mounted with10 µg/ml DAPI. Images were merged using Adobe Photoshop 5.0 software.

Immunoelectron microscopy was carried out with purified *P. falciparum* schizonts as described previously [31]. Briefly, mature-stage parasitized cells were fixed with 1% paraformaldehyde and 0.1% glutaraldehyde, and reacted with rabbit anti-rPfRH5. Anti-rabbit Ig coupled to 10 nm gold particles was used to detect immuno-reactivity.

### Erythrocyte binding assays (EBA)

Schizont stage parasites were purified by centrifugation on 40%/70%/90% Percoll/sorbitol gradients, as described.[32]. To make culture supernatants, purified schizonts were returned to *in-vitro* culture at 2.5×10^7^ parasites/ml with [^35^S] methionine/cysteine (ICN) and allowed to rupture overnight. Cells were harvested by centrifugation and radio-labeled supernatants were stored at −80°C. To perform erythrocyte binding assays (EBAs), radio-labeled culture supernatant was rotated with the appropriate erythrocytes at room temperature for 2 h. The erythrocytes were then washed three times with RPMI 1640 incomplete medium, layered on dibutylphthalate (Sigma), and centrifuged at 6,000×*g* for 1 min. The supernatant and oil were removed by aspiration. Bound parasite proteins were eluted from the erythrocytes with 1.5 M NaCl and the eluate was used for immunoprecipitation by anti-RH5 and anti-EBA-175 antibodies.

For enzymatic treatment, erythrocytes were treated at 1×10^8^ cells/ml in RPMI with three different concentrations of trypsin (Sigma): Low trypsin (LT): 0.5 mg/ml; Moderate trypsin (MT): 1.5 mg/ml trypsin and High trypsin (HT): 10 mg/ml. Cells were also treated with 0.025 U/ml neuraminidase (*Vibrio cholerae*; Roche), or 2 mg/ml chymotrypsin (Sigma) at 37°C for 1 h. Cells treated with trypsin were then washed and treated with 0.5 mg/ml soybean trypsin inhibitor (Sigma) for 15 min at room temperature, as described earlier [14]. The efficacy of each enzyme treatment was assessed in the Laboratory of Immunohematology, New York Blood Center, by assaying for loss of RBC agglutinability, using a panel of monoclonal antibodies against suitable antigenic determinants on different blood group proteins.

In EBAs with rRH5, 0.6 µmoles of recombinant protein in 100 µl of PBS at pH 7.4 was incubated with 100 µl of packed erythrocytes, wild type and enzyme treated as described above, at 37°C for 2 h. Thereafter, the EBA was followed as described above. rRH5 was detected in the eluate by immunoprecipitation, followed by immuno-blotting (to minimize carryover from the IgG) with the anti-RH5 antibodies, using a kit from Genscript and following manufacturer's instructions.

### Labeling of rRH5 for use in binding experiments and generation of binding profiles

Recombinant RH5 protein was labeled with Rhodamine Red (Fluo-reporter labeling kit, Molecular Probes), according to the manufacturer's direction. The rhodamine-red has a reactive succinimidyl ester moiety, which reacts with the primary amines of proteins to form a covalent conjugate. The labeled protein was dialyzed exhaustively against PBS, pH 7.4, for 3 days, changing the buffer several times. A calibration standard plot (which proved linear) was generated with 1 to 60 nmoles rhodamine-labeled rRh5, covering the concentration range of the binding assays. rRH5 was used in binding experiments with freshly prepared resealed red cell ghosts (to avoid fluorescence quenching by hemoglobin). 25 µl RBC ghosts in 100 µl PBS (pH 7.4) were incubated with different amounts of labeled rRH5 for 2 hr at RT and washed 4 times with 5 mM Na-Phosphate buffer, 1 mm EDTA pH 7.4 to remove unbound protein. The ghosts were then dissolved with 0.1% SDS. The amount of rRH5 bound to RBC ghosts was determined from the fluorescence emission intensity at 590 nm, with excitation at 570 nm, in an RF 5301 spectrofluorimeter.

### Competition binding assays

2 µM Rhodamine-rRh5 was incubated with 25 µl of packed RBC ghosts in a total volume of 200 µl of PBS at pH 7 with increasing amounts of unlabeled rRH5, to give final concentrations from 0.1 to 400 µM .The mixture was incubated at RT for 2 h and the cells were washed 4 times with 5 mM Na-Phosphate buffer, 1 mmEDTA, pH 7.4 to remove unbound protein. The ghosts were then dissolved with 0.1% SDS and bound labeled rRH5 was determined by spectrofluorimetry as above.

### Overlay assays

The overlay protein-binding assay was done using rRH5 or native parasite supernatant (overnight) and erythrocyte ghosts. Erythrocyte ghosts were prepared by hypotonic lysis as described before [33]. Ghost proteins (30 µg/lane, corresponding to ∼5×10^7^ cells) were separated by 10% SDS–PAGE and transferred onto nitrocellulose. The membrane was stained with Ponceau stain, cut into strips and blocked with 10% milk powder in phosphate-buffered saline/0.1% Tween-20 (PBST) for 1 h. After washing with PBST three times, 5 min per wash, the strips were incubated overnight at 4°C with parasite culture supernatant (Dd2) or with rRH5. The strips were washed five times (5 min each wash) before antigen detection proceeded as outlined above for immuno-blotting. To find the positions of the glycophorins on the blot, strips were treated with anti-GPA/GPB (E3 from Sigma) or anti-GPC (E5 from Sigma).

### Antibody-Mediated blocking of binding of rRH5 to RBCs

IgG was purified from either pre-immune serum or anti-RH5 serum using Immuno-pure Binding/Elution Buffer System (Protein G) from Pierce. To assay for inhibition of erythrocyte binding of the rPfRH5 protein, 0.6 µmoles of rRH5 was incubated with 100 µl of packed normal erythrocytes in the presence of anti-RH5 total IgG at a final concentration ranging from 0 to10 µg/100 µl rabbit IgG at 37°C for 1 h. Thereafter, the EBA was followed as described above.

### Invasion assays

Schizont stage parasites were purified as above, and placed into *in-vitro* culture with the appropriate target erythrocytes (untreated or treated with enzymes, as described above). Ring stage parasites were counted following a 20-h incubation. All invasion assays were performed in triplicate, and at least 1,000 erythrocytes were counted per assay. IgG was purified from either pre-immune serum or anti-RH5 serum as indicated above. After elution from the Protein G column, IgG was dialyzed against RPMI and added to invasion assays at a concentration of 0.5 mg/ml. Invasion in the presence of specific RH5 antibodies was related to invasion in the presence of nonspecific antibodies from pre-immune serum.

## References

[1] Guinovart C, Navia MM, Tanner M, Alonso PL (2006). Malaria: burden of disease.. Current molecular medicine.

[2] Gratzer WB, Dluzewski AR (1993). The red blood cell and malaria parasite invasion.. Semin Hematol.

[3] Sim BK, Chitnis CE, Wasniowska K, Hadley TJ, Miller LH (1994). Receptor and ligand domains for invasion of erythrocytes by Plasmodium falciparum.. Science.

[4] Dolan SA, Proctor JL, Alling DW, Okubo Y, Wellems TE (1994). Glycophorin B as an EBA-175 independent Plasmodium falciparum receptor of human erythrocytes.. Mol Biochem Parasitol.

[5] Lobo CA, Rodriguez M, Reid M, Lustigman S (2003). Glycophorin C is the receptor for the Plasmodium falciparum erythrocyte binding ligand PfEBP-2 (baebl).. Blood.

[6] Maier AG, Duraisingh MT, Reeder JC, Patel SS, Kazura JW (2003). Plasmodium falciparum erythrocyte invasion through glycophorin C and selection for Gerbich negativity in human populations.. Nat Med.

[7] Rayner JC, Vargas-Serrato E, Huber CS, Galinski MR, Barnwell JW (2001). A Plasmodium falciparum homologue of Plasmodium vivax reticulocyte binding protein (PvRBP1) defines a trypsin-resistant erythrocyte invasion pathway.. J Exp Med.

[8] Duraisingh MT, Triglia T, Ralph SA, Rayner JC, Barnwell JW (2003). Phenotypic variation of Plasmodium falciparum merozoite proteins directs receptor targeting for invasion of human erythrocytes.. Embo J.

[9] Gilberger TW, Thompson JK, Triglia T, Good RT, Duraisingh MT (2003). A novel erythrocyte binding antigen-175 paralogue from Plasmodium falciparum defines a new trypsin-resistant receptor on human erythrocytes.. J Biol Chem.

[10] Dolan SA, Miller LH, Wellems TE (1990). Evidence for a switching mechanism in the invasion of erythrocytes by Plasmodium falciparum.. J Clin Invest.

[11] Mitchell GH, Hadley TJ, McGinniss MH, Klotz FW, Miller LH (1986). Invasion of erythrocytes by Plasmodium falciparum malaria parasites: evidence for receptor heterogeneity and two receptors.. Blood.

[12] Binks RH, Conway DJ (1999). The major allelic dimorphisms in four Plasmodium falciparum merozoite proteins are not associated with alternative pathways of erythrocyte invasion.. Mol Biochem Parasitol.

[13] Okoyeh JN, Pillai CR, Chitnis CE (1999). Plasmodium falciparum field isolates commonly use erythrocyte invasion pathways that are independent of sialic acid residues of glycophorin A.. Infect Immun.

[14] Lobo CA, de Frazao K, Rodriguez M, Reid M, Zalis M (2004). Invasion profiles of Brazilian field isolates of Plasmodium falciparum: phenotypic and genotypic analyses.. Infect Immun.

[15] Baum J, Pinder M, Conway DJ (2003). Erythrocyte invasion phenotypes of Plasmodium falciparum in The Gambia.. Infect Immun.

[16] Jennings CV, Ahouidi AD, Zilversmit M, Bei AK, Rayner J (2007). Molecular analysis of erythrocyte invasion in Plasmodium falciparum isolates from Senegal.. Infect Immun.

[17] Adams JH, Blair PL, Kaneko O, Peterson DS (2001). An expanding ebl family of Plasmodium falciparum.. Trends Parasitol.

[18] Chitnis CE, Miller LH (1994). Identification of the erythrocyte binding domains of Plasmodium vivax and Plasmodium knowlesi proteins involved in erythrocyte invasion.. J Exp Med.

[19] Khan SM, Jarra W, Preiser PR (2001). The 235 kDa rhoptry protein of Plasmodium (yoelii) yoelii: function at the junction.. Mol Biochem Parasitol.

[20] Galinski MR, Medina CC, Ingravallo P, Barnwell JW (1992). A reticulocyte-binding protein complex of Plasmodium vivax merozoites.. Cell.

[21] Cowman AF, Crabb BS (2006). Invasion of red blood cells by malaria parasites.. Cell.

[22] Iyer J, Gruner AC, Renia L, Snounou G, Preiser PR (2007). Invasion of host cells by malaria parasites: a tale of two protein families.. Mol Microbiol.

[23] Gaur D, Singh S, Singh S, Jiang L, Diouf A (2007). Recombinant Plasmodium falciparum reticulocyte homology protein 4 binds to erythrocytes and blocks invasion.. Proc Natl Acad Sci U S A.

[24] Taylor HM, Grainger M, Holder AA (2002). Variation in the expression of a Plasmodium falciparum protein family implicated in erythrocyte invasion.. Infect Immun.

[25] Rayner JC, Galinski MR, Ingravallo P, Barnwell JW (2000). Two Plasmodium falciparum genes express merozoite proteins that are related to Plasmodium vivax and Plasmodium yoelii adhesive proteins involved in host cell selection and invasion.. Proc Natl Acad Sci U S A.

[26] Sam-Yellowe TY, Shio H, Perkins ME (1988). Secretion of Plasmodium falciparum rhoptry protein into the plasma membrane of host erythrocytes.. J Cell Biol.

[27] Reid ME, Lomas-Francis C (1997).

[28] Lobo CA, Rodriguez M, Struchiner CJ, Zalis MG, Lustigman S (2006). Associations between defined polymorphic variants in the PfRH ligand family and the invasion pathways used by P. falciparum field isolates from Brazil.. Mol Biochem Parasitol.

[29] Trager W, Jensen JB (1976). Human malaria parasites in continuous culture.. Science.

[30] Su XZ, Carucci DJ, Wellems TE (1998). Plasmodium falciparum: parasite typing by using a multicopy microsatellite marker, PfRRM.. Exp Parasitol.

[31] Lobo CA, Rodriguez M, Hou G, Perkins M, Oskov Y (2003). Characterization of PfRhop148, a novel rhoptry protein of Plasmodium falciparum.. Mol Biochem Parasitol.

[32] Wahlgren M, Berzins K, Perlmann P, Persson M (1983). Characterization of the humoral immune response in Plasmodium falciparum malaria. II. IgG subclass levels of anti-P. falciparum antibodies in different sera.. Clinical and experimental immunology.

[33] Fairbanks G, Steck TL, Wallach DF (1971). Electrophoretic analysis of the major polypeptides of the human erythrocyte membrane.. Biochemistry.

