# Correction: PfRH5: A Novel Reticulocyte-Binding Family Homolog of *Plasmodium falciparum* that Binds to the Erythrocyte, and an Investigation of Its Receptor

**DOI:** 10.1371/annotation/dde6c172-c9c3-43bb-8fc3-db54613d4424

**Published:** 2008-11-05

**Authors:** Marilis Rodriguez, Sara Lustigman, Estrella Montero, Yelena Oksov, Cheryl A. Lobo

The original figures in this article were adversely affected by imaging artefacts as a result of low-resolution scanning that diminished the quality of the data. High-resolution images corresponding to Figures 1, 3, 4, and 6 can be found here. The new images are fully consistent with the original images and completely support the conclusions in the article.

Figure 1 high-resolution version (4.6 MB TIF)

Figure 3 high-resolution version (3.6 MB TIF)

Figure 4 high-resolution version (2.9 MB TIF)

Figure 6 high-resolution version (4.5 MB TIF)

