# Supplementary figures and images for: Correction: PfRH5: A Novel Reticulocyte-Binding Family Homolog of Plasmodium falciparum that Binds to the Erythrocyte, and an Investigation of Its Receptor
Source: PLoS One. 2008 Nov 5;3(11):10.1371/annotation/dde6c172-c9c3-43bb-8fc3-db54613d4424. doi: 10.1371/annotation/dde6c172-c9c3-43bb-8fc3-db54613d4424 (PMC2586816; doi:10.1371/annotation/dde6c172-c9c3-43bb-8fc3-db54613d4424)

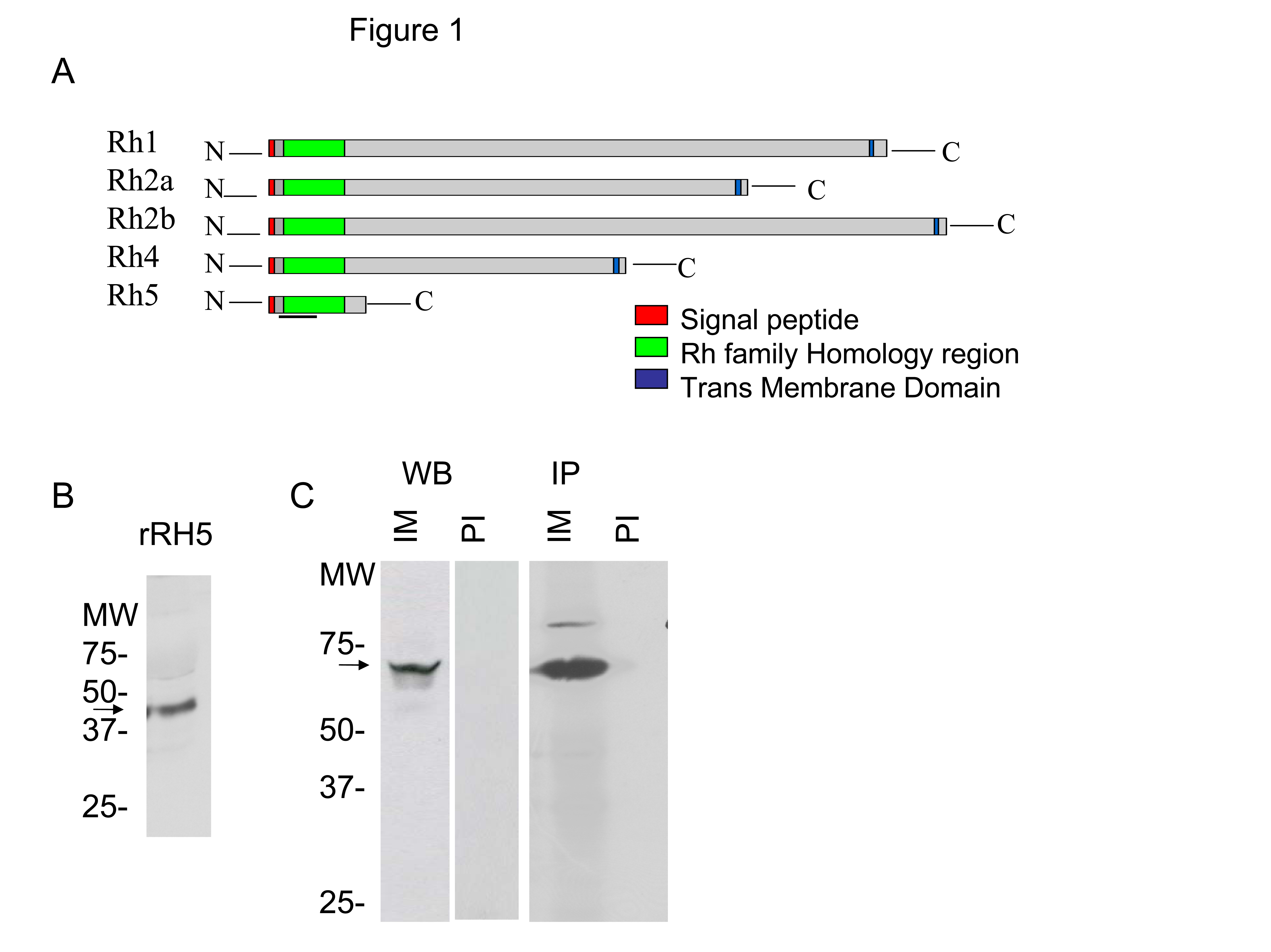

Supplement: Figure 1 high-resolution version — (4.6 MB TIF) [file pone.dde6c172-c9c3-43bb-8fc3-db54613d4424.s001.tif]

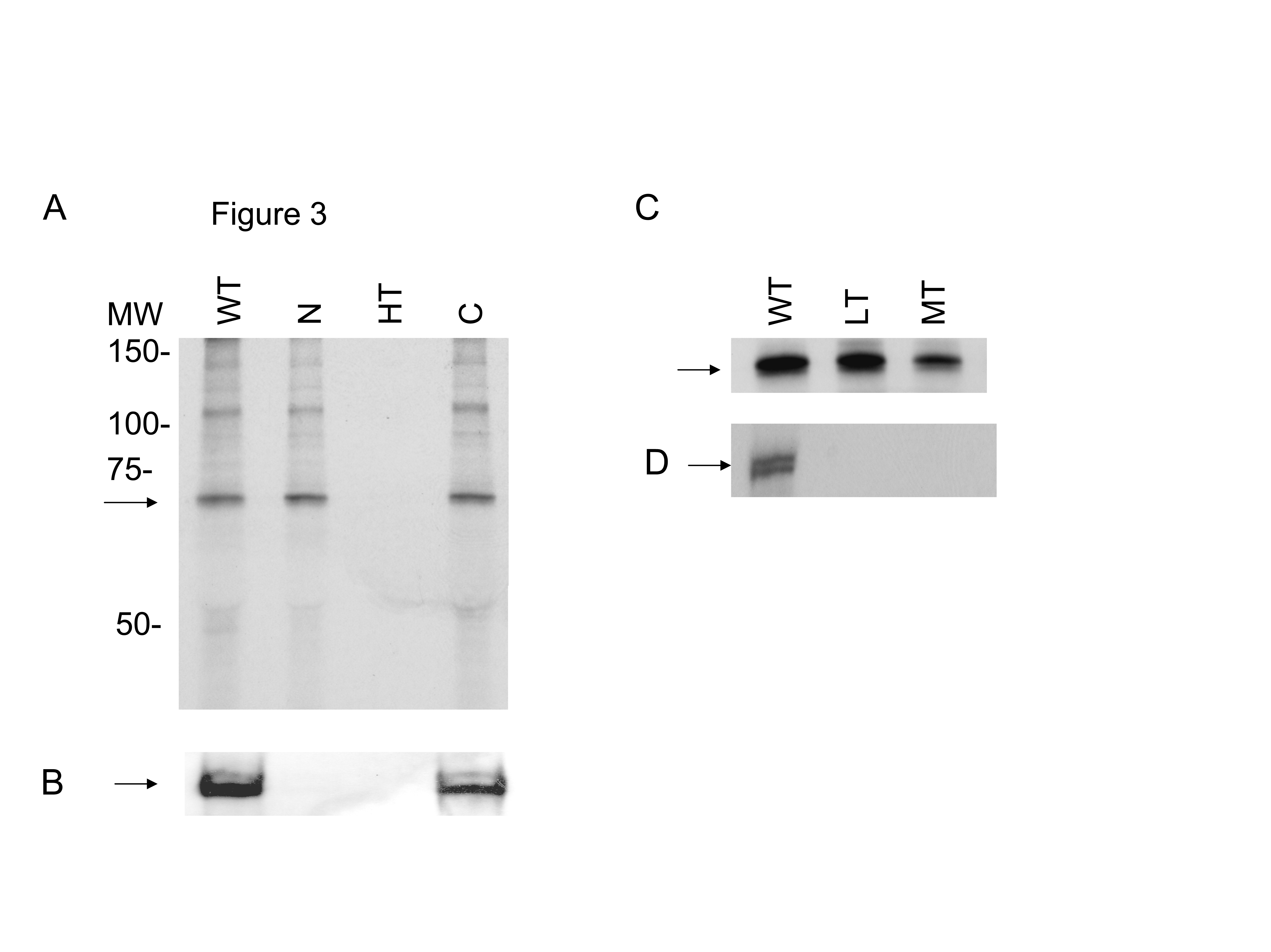

Supplement: Figure 3 high-resolution version — (3.6 MB TIF) [file pone.dde6c172-c9c3-43bb-8fc3-db54613d4424.s002.tif]

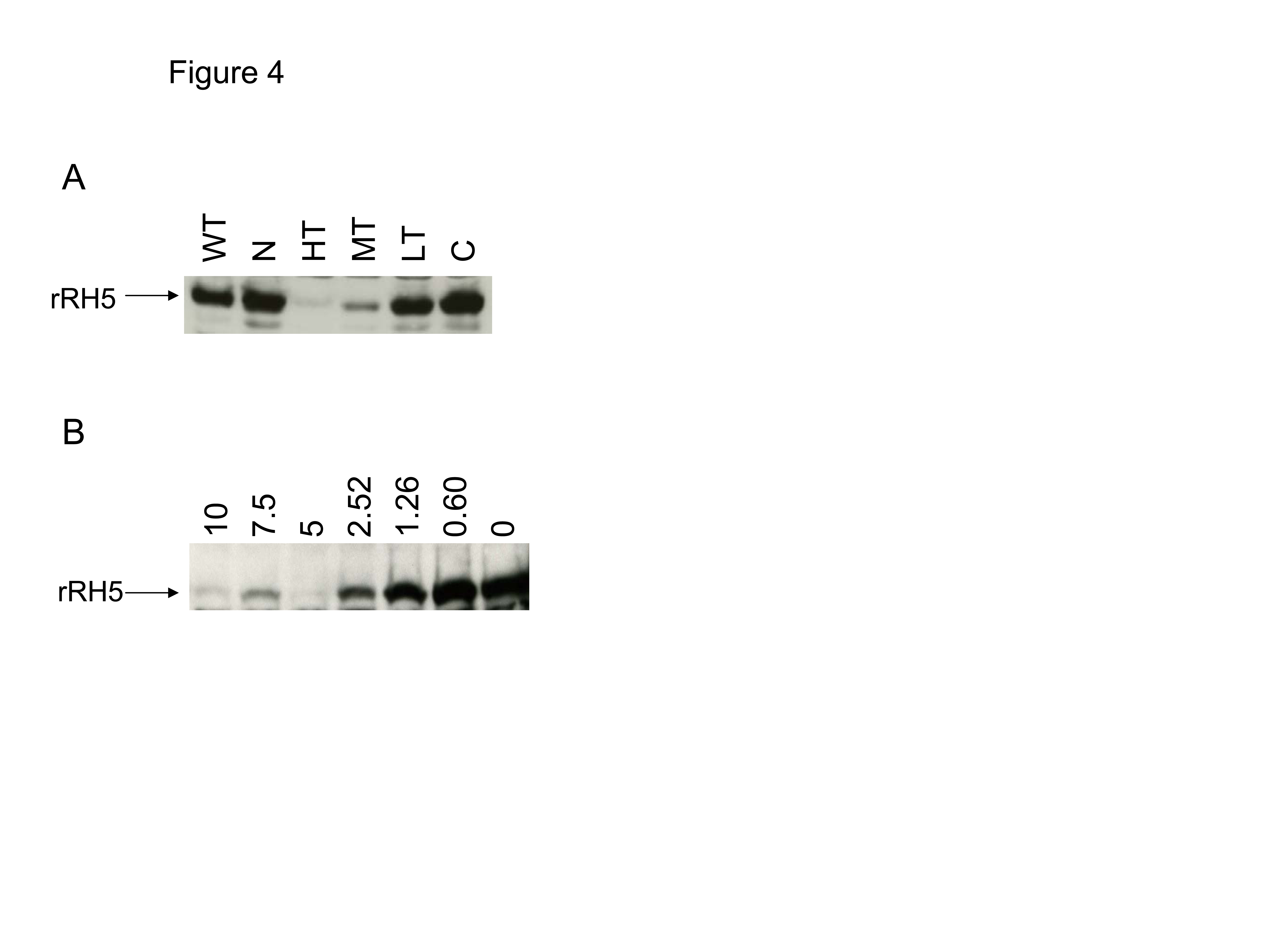

Supplement: Figure 4 high-resolution version — (2.9 MB TIF) [file pone.dde6c172-c9c3-43bb-8fc3-db54613d4424.s003.tif]

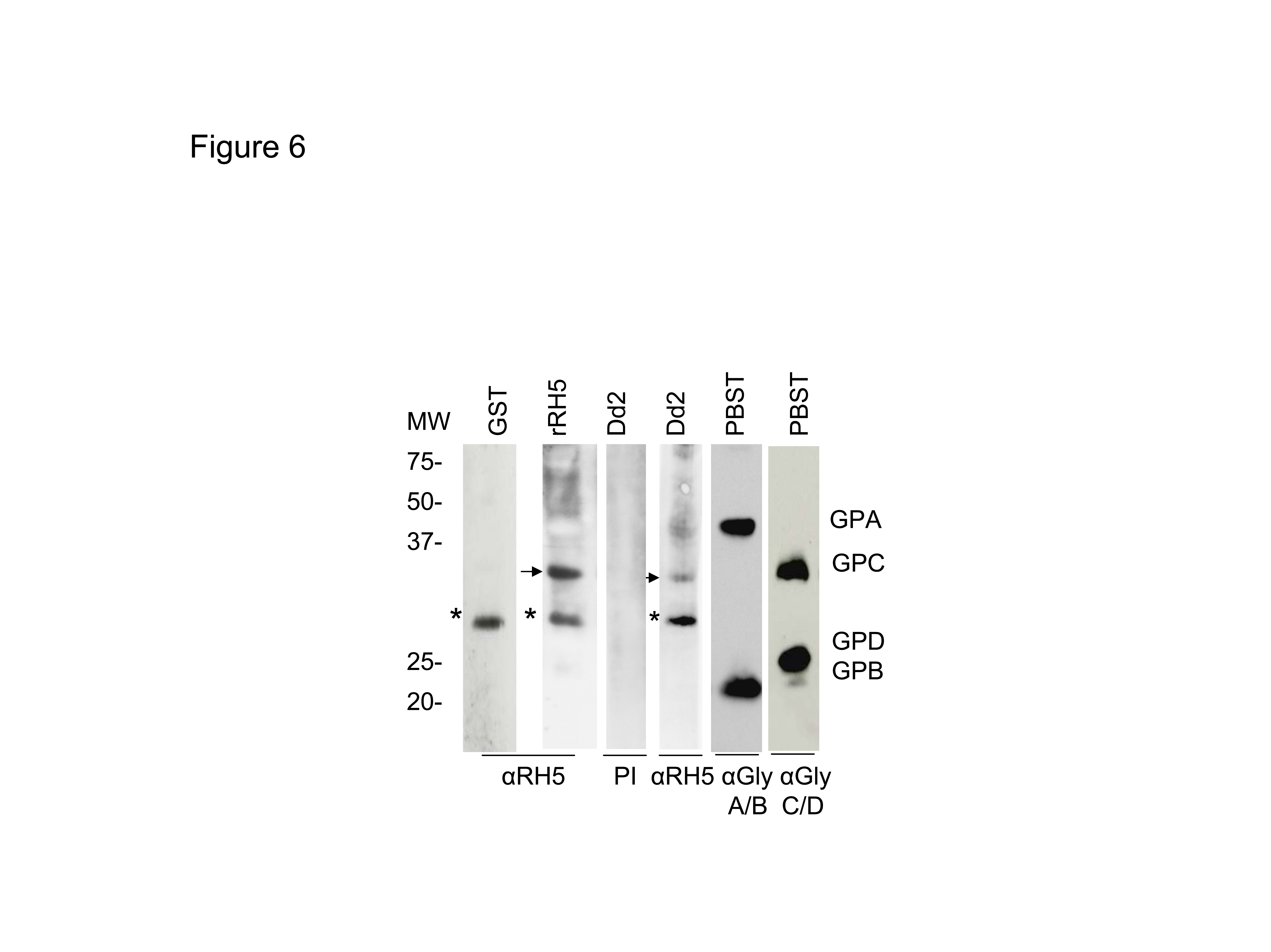

Supplement: Figure 6 high-resolution version — (4.5 MB TIF) [file pone.dde6c172-c9c3-43bb-8fc3-db54613d4424.s004.tif]
